# A Useful Tool As a Medical Checkup in a General Population—Bioelectrical Impedance Analysis

**DOI:** 10.3389/fcvm.2017.00003

**Published:** 2017-02-02

**Authors:** Mika Enomoto, Hisashi Adachi, Ako Fukami, Eita Kumagai, Sachiko Nakamura, Yume Nohara, Shoko Kono, Erika Nakao, Nagisa Morikawa, Tomoko Tsuru, Akiko Sakaue, Yoshihiro Fukumoto

**Affiliations:** ^1^Division of Cardiovascular Medicine, Department of Internal Medicine, Kurume University School of Medicine, Kurume, Japan; ^2^Department of Community Medicine, Kurume University School of Medicine, Kurume, Japan

**Keywords:** visceral fat area, metabolic syndrome, health examination, general population, epidemiological study

## Abstract

Accumulation of visceral fat leads to metabolic syndrome and increases risks of cerebro-cardiovascular diseases, which should be recognized and improved at the early stage in general population. Accurate measurement of visceral fat area (VFA) is commonly performed by the abdominal cross-sectional image measured by computed tomography scan, which is, however, limited due to the radiation exposure. The bioelectrical impedance analysis (OMRON, HDS-2000 DUALSCAN^R^) has been recently developed to measure VFA, which is more easily accessible modality. In the present study, we investigated the clinical usefulness of DUALSCAN^R^ in 226 subjects who received health examination, including blood chemistries, electrocardiography, cardio, and carotid ultrasonography. VFA was measured within only just 5 min. Average of VFA was 83.5 ± 36.3 cm^2^ in men, and 64.8 ± 28.0 cm^2^ in women, which was correlated to weight (*r* = 0.7404, *p* < 0.0001), body mass index (BMI) (*r* = 0.7320, *p* < 0.0001), and waist circumstance (*r* = 0.7393, *p* < 0.0001). In multivariate analyses, VFA was significantly associated with weight (*p* < 0.0001), BMI (*p* < 0.0001), and waist circumstance (*p* < 0.0001). Compared to the group of smaller waist and normal BMI, VFA was significantly increased (*p* < 0.0001) in the group of larger waist and obese subjects. In conclusion, these results indicated that DUALSCAN^R^ is useful to measure VFA easily in general population, even in a large number of subjects.

## Introduction

Obesity has been rapidly increasing over the last decades all over the world (1), which is a serious concern and should be recognized and improved at the early stage in general population, especially in the aspects of the increased risk of cardiovascular diseases as primary prevention (2). It is simple for obesity assessment to measure body mass index (BMI), waist circumference, or waist-to-hip ratio. However, it is clinically important to distinguish visceral from subcutaneous adipose tissues by using imaging techniques, such as magnetic resonance imaging (MRI) or computed tomography (CT) scan (3, 4), because visceral fat accumulation was found to be specifically associated with the metabolic alterations of obesity, both in men and women (5). Serial-slice CT is able to evaluate the visceral adipose tissue (VAT) in detail (6). Dual-energy X-ray absorptiometry (DEXA) is usually used to measure total body fat, which has been also used to measure central abdominal fat (7). It has been reported that visceral fat measured by CT scan has a strong correlation with central abdominal fat measured by DEXA (7–14).

However, these methods are limited in the clinical use, due to the radiation exposure and the cost of techniques, which is not suitable to general health checkup examination. Where more easily accessible modality at lower cost should be developed, the bioelectrical impedance analysis (BIA) has been recently developed, which is used in population and clinical studies as a technique to estimate body composition (15–19). Unno and colleagues (15) found that VFA estimated by BIA is useful for detection of metabolic syndrome (MetS), as consistently reported by other studies (16–20).

Therefore, in the present study, we aimed to examine whether the new method of BIA is useful to measure VFA in Japanese general population.

## Subjects and Methods

### Study Population

A total of 226 subjects (87 males and 139 females: aged 40–90 years) received a population-based health examination in Uku town, a fishing community in southwestern Japan in 2011. This town is an isolated island in Sasebo city, located in Nagasaki prefecture, and the total population is about 3,700. A detailed content of the recent survey in the same district was previously described (21, 22).

### Data Collection

Height and weight were measured, and BMI was calculated as weight (kilograms) divided by the square of height (square meters) as an index of the presence or absence of obesity. Waist circumference was measured at the level of the umbilicus in a standing position. Blood pressure (BP) was measured twice with the subjects in the sitting (first) and supine (second) position. Vigorous physical activity and smoking were avoided for at least 30 min before BP measurements. The second BP with the fifth phase diastolic pressure was used for analysis.

Blood was drawn from the antecubital vein for determinations of lipids profiles [total cholesterol, high-density lipoprotein cholesterol (HDL-c), low-density lipoprotein cholesterol (LDL-c), and triglycerides], creatinine, uric acid (UA), fasting plasma glucose (FPG), glycated hemoglobin A_1c_ [HbA_1c_(NGSP)], and high-sensitivity C-reactive protein (hs-CRP) in the morning after a 12-h fast. Fasting blood samples were centrifuged within 1 h after the collection. Estimated glomerular filtration rate (eGFR) was calculated using the modification of diet in renal disease (MDRD) study equation modified with a Japanese coefficient (23).

All subjects underwent the new abdominal BIA method (OMRON, HDS-2000 DUALSCAN^R^) to estimate VFA in the morning after a 12-h fast. The voltage occurring at the flank to the flow of current between the umbilicus and the back correlates significantly with VFA and is unaffected by subcutaneous fat area. The voltage becomes larger as VFA even in the subjects with the same waist circumference because the electric resistance of intra-abdominal fat is greater than that of fat-free mass, and the density of the equipotential lines between two electrodes becomes denser. Using this method, we measured VFA of individuals within 5 min.

C-IMT of the common carotid artery was determined by using duplex ultrasonography (Sonosite “TITAN,” ALOKA) with a 10-MHz transducer in the supine position. Longitudinal B-mode images at the diastolic phase of the cardiac cycle were recorded by a single trained technician who was blinded to the subjects’ background. We measured the only far site of the wall of c-IMT. The images were magnified and measured on the screen and printed with a high-resolution line recorder (LSR-100A, Toshiba). We measured c-IMT according to the originally described method published in Circulation (24). Briefly, the c-IMT defined by Pignoli et al. (24) was measured as the distance from the leading edge of the first echogenic line to the leading edge of the second echogenic line. The first line represented the lumen–intimal interface; the collagen-containing upper layer of the tunica adventitia formed the second line. At each longitudinal projection, the site of the greatest thickness, including plaque, was sought along the arterial walls nearest and farthest from the skin from the common carotid artery to the internal carotid artery. Three determinations of c-IMT of one artery were conducted at the site of the greatest thickness and at two other points, 1 cm upstream and 1 cm downstream from this site. The averaged value among the six IMTs (three from the left and three from the right) was used as the representative value for each individual.

This study was approved by the mayor, by the welfare department of Uku town, and by the Research Ethics Committee of the Kurume University School of Medicine (Process numbers 2284), approved the study in conformity with the principles embodied in the declaration of Helsinki. All participants were informed about research procedures and risks before signing an informed consent.

### Statistical Analysis

Results were presented as means ± SDs. Because of skewed distributions, natural logarithmic (ln) transformations were performed for triglycerides and hs-CRP. Log-transformed values were reconverted to antilogarithm forms in the tables. The medications for hypertension, dyslipidemia, and diabetes mellitus were coded as dummy variables. Gender, smoking habits, and alcohol intake were also coded as dummy variables. First, we performed univariate analysis for VFA levels as dependent variable. Second, multivariate analysis after adjustments for age and sex was performed for correlates of VFA levels. Finally, comparisons of various parameters between VFA levels (VFA < 100 cm^2^ vs. VFA ≥ 100 cm^2^) was performed. Statistical significance was defined as *p* < 0.05. All statistical analyses were performed using the SAS system (Release 9.3, SAS Institute, Cary, NC, USA).

## Results

**Figure 1:**
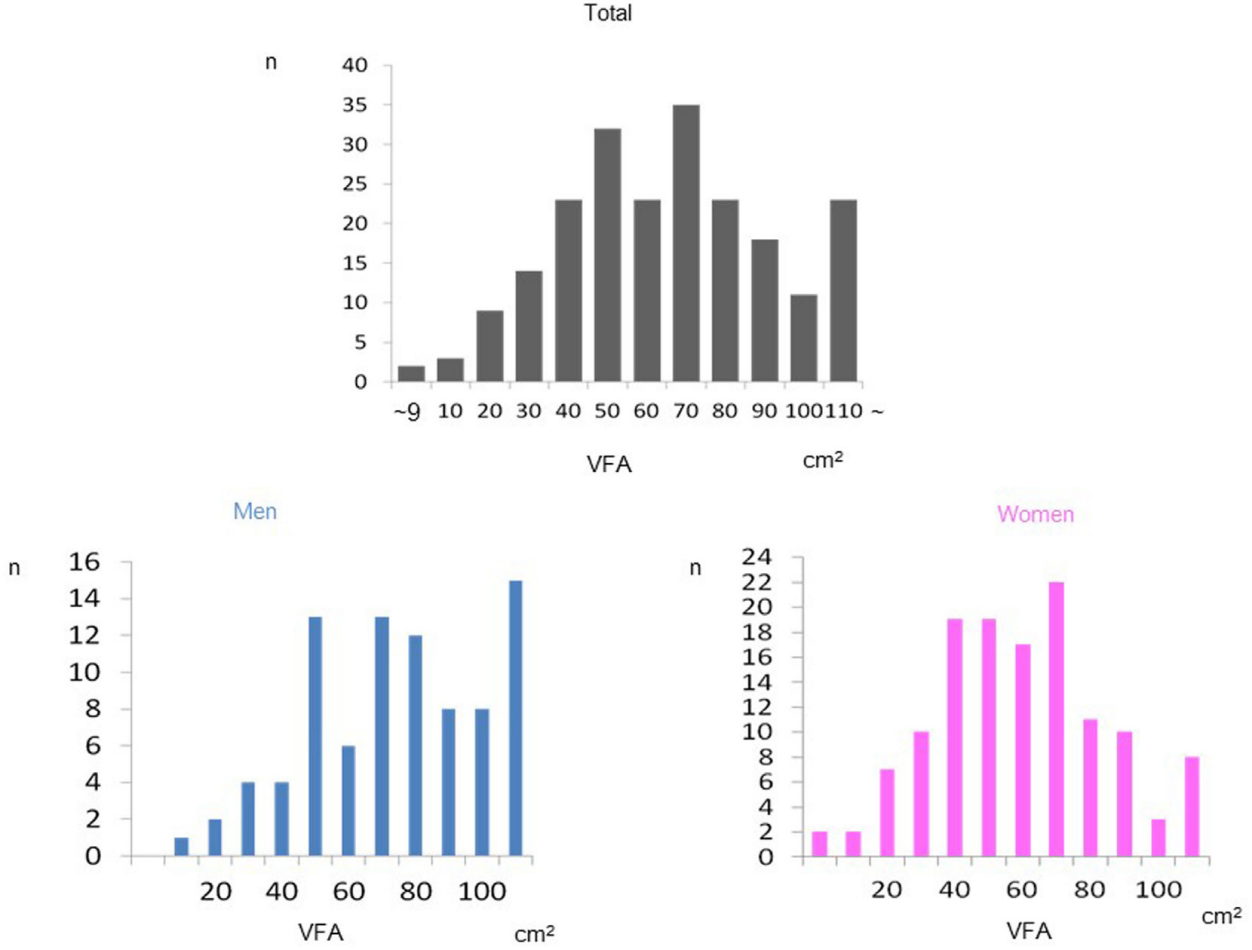
**The prevalence of VFA in total and both sexes were shown**.

**Figure 2:**
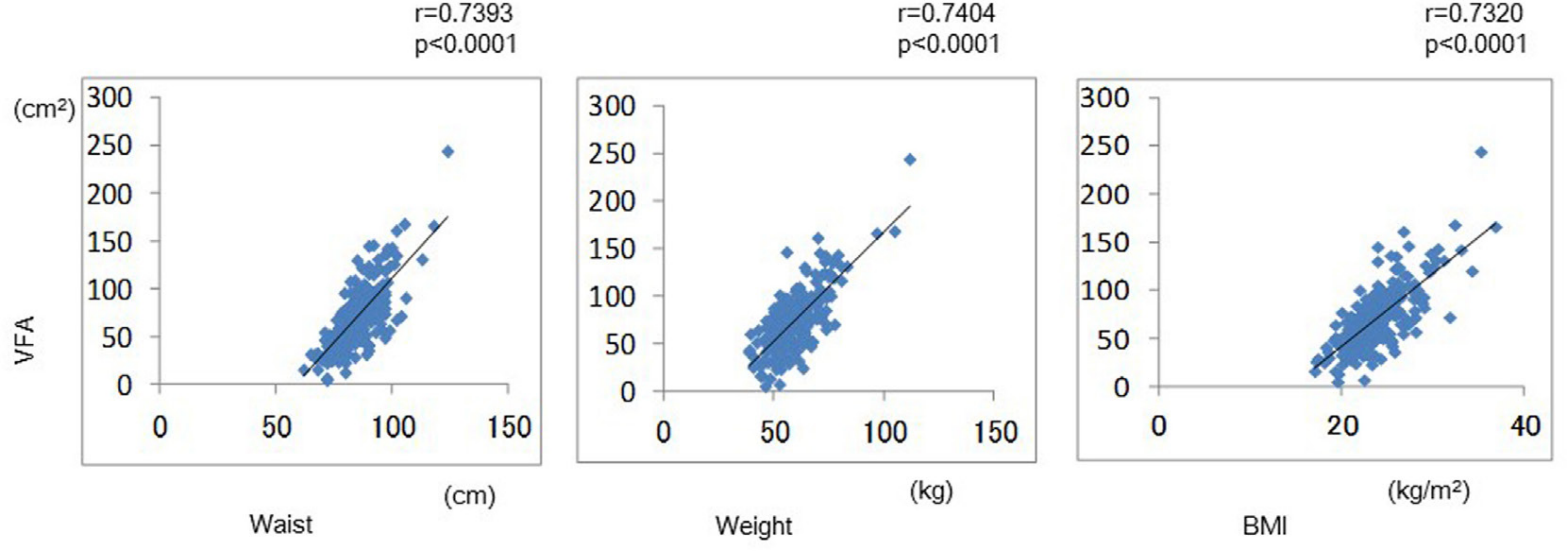
**The correlation between VFA values and waist, weight, and body mass index were demonstrated**.

Demographic and clinical characteristics of the enrolled study subjects are presented in Table 1. Mean values of VFA were 83.5 cm^2^ in men and 64.8 cm^2^ in women, respectively, indicating that most of the subjects were non-obese. Mean VFA values of most subjects were within normal range. The prevalence of VFA in total and both genders was shown in Figure 1. VFA levels had a normal distribution in both genders. The association between VFA values and waist, weight, and BMI were demonstrated in Figure 2. Strong association between VFA and waist (*r* = 0.739, *p* < 0.0001), VFA and weight (*r* = 0.740, *p* < 0.0001), and VFA and BMI (*r* = 0.732, *p* < 0.0001) were observed.

**Table 1:** **Characteristics of study subjects**.

Parameters	Males	Females	Total
Number	87	139	226
Age (years)	68.3 ± 8.2	66.4 ± 10.6	67.1 ± 9.8
Weight (kg)	64.5 ± 10.5	54.2 ± 9.0	58.2 ± 10.8
BMI (kg/m^2^)	24.0 ± 3.0	23.7 ± 3.5	23.8 ± 3.3
Waist (cm)	86.6 ± 8.5	83.9 ± 9.9	85.0 ± 9.5
Systolic BP (mmHg)	135.4 ± 17.4	139.1 ± 20.4	137.7 ± 19.4
Diastolic BP (mmHg)	86.8 ± 8.9	84.3 ± 9.9	85.3 ± 9.6
Total cholesterol (mg/dl)	194.7 ± 43.8	207.6 ± 33.9	202.7 ± 38.4
HDL-cholesterol (mg/dl)	55.1 ± 14.3	65.4 ± 14.7	63.7 ± 36.4
LDL-cholesterol (mg/dl)	115.1 ± 34.3	121.1 ± 29.7	118.8 ± 31.6
Triglycerides (mg/dl)^a^ (min–max)	93.0 (34–399)	75.9 (37–250)	82.2 (34–399)
FPG (mg/dl)	110.7 ± 27.4	93.6 ± 10.0	100.1 ± 20.4
HbA_1c_ (NGSP) (%)	5.7 ± 0.8	5.5 ± 0.5	5.5 ± 0.7
eGFR (ml/min/1.73 m^2^)	75.1 ± 17.7	72.8 ± 16.6	73.7 ± 17.1
Uric acid (mg/dl)	6.0 ± 1.2	4.7 ± 1.1	5.2 ± 1.3
hs-CRP (mg/dl)^a^ (min–max)	0.050 (0.004–3.830)	0.037 (0.004–4.940)	0.042 (0.004–4.940)
C-IMT (mm)	0.8 ± 0.2	0.7 ± 0.1	0.8 ± 0.2
Hypertensive medication (%)	52.9	39.6	44.7
Diabetic medication (%)	14.9	5.0	8.9
Dyslipidemic medication (%)	21.8	28.8	26.1
Habitual smoking (%)	23.0	2.2	10.2
Alcohol intake (%)	54.0	6.5	24.8
VFA (cm^2^)	83.5 ± 36.3	64.8 ± 28.0	72.3 ± 32.8

*Data are means ± SDs, geometric mean, range, or percent*.

*VFA, visceral fat area; BMI, body mass index; BP, blood pressure; HDL, high-density lipoprotein; LDL, low-density lipoprotein; FPG, fasting plasma glucose; eGFR, estimated glomerular filtration rate; hs-CRP, high sensitivity C-reactive protein; C-IMT, carotid intima-media thickness*.

*^a^Log-transformed values were used for the analysis*.

**Figure 3:**
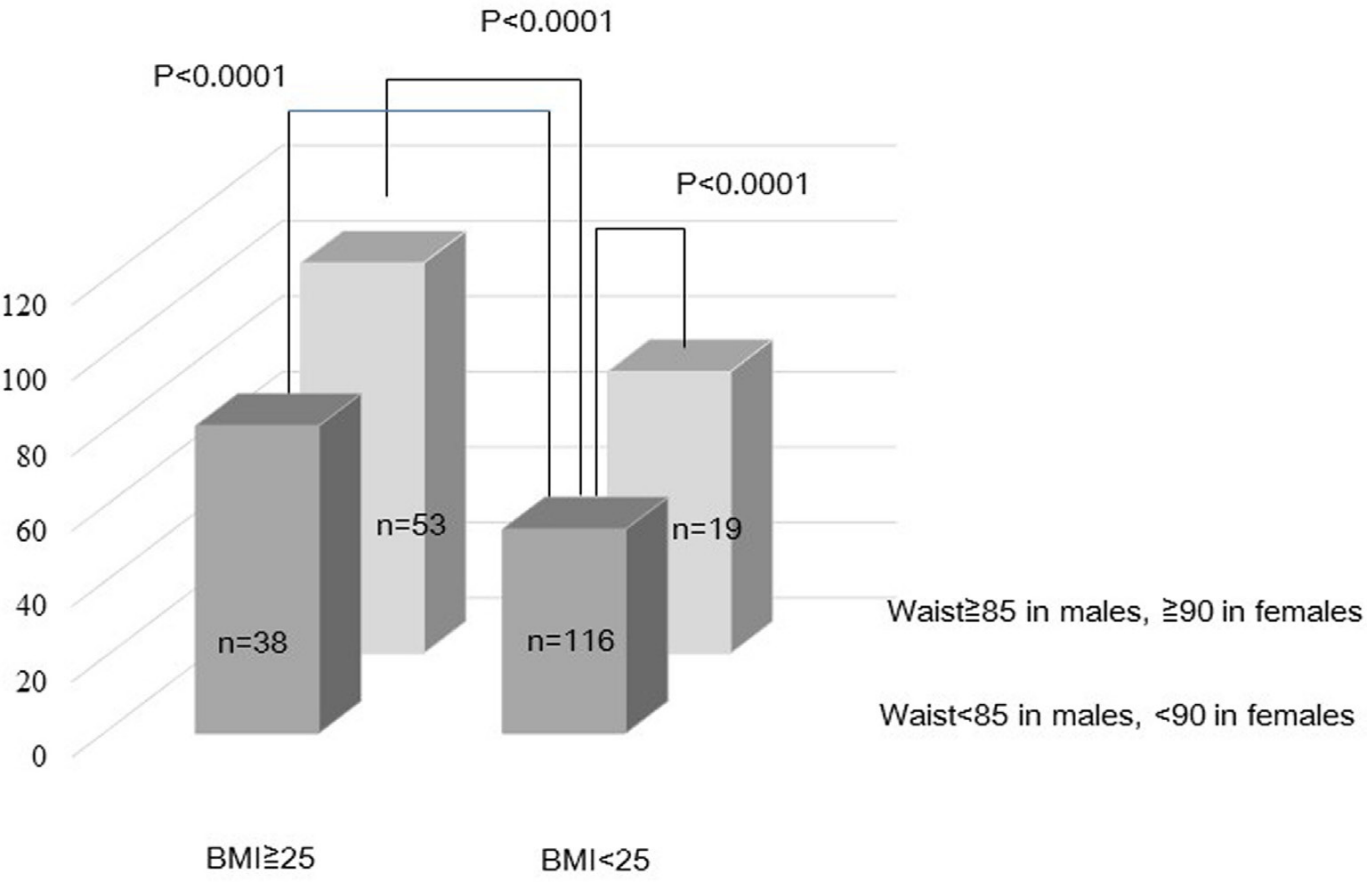
**Relationship between VFA, waist, and body mass index**.

Univariate analysis for VFA levels as dependent variable stratified by gender was shown in Table 2. Weight (*p* < 0.0001), BMI (*p* < 0.0001), waist (*p* < 0.0001), triglycerides (*p* < 0.0001), FPG (*p* < 0.01), hypertensive medication (*p* < 0.05), dyslipidemic medication (*p* < 0.05), and alcohol intake (*p* < 0.01) were significantly associated with VFA areas in males. Age (*p* < 0.01), weight (*p* < 0.0001), BMI (*p* < 0.0001), waist (*p* < 0.0001), systolic BP (*p* < 0.05), HDL-c (*p* < 0.0001; inversely), triglycerides (*p* < 0.01), FPG (*p* < 0.01), HbA_1c_ (*p* < 0.01), UA (*p* < 0.0001), hs-CRP (*p* < 0.05), c-IMT (*p* < 0.01), hypertensive medication (*p* < 0.05), diabetic medication (*p* < 0.01), and dyslipidemic medication (*p* < 0.001) were significantly associated with VFA areas in females. Table 3 shows the multivariate analysis adjusted for age for VFA levels as dependent variable. Weight (*p* < 0.0001), BMI (*p* < 0.0001), waist (*p* < 0.0001), triglycerides (*p* < 0.0001), FPG (*p* < 0.05), hypertensive medication (*p* < 0.05), dyslipidemic medication (*p* < 0.05), and alcohol intake (*p* < 0.01) in males. Weight (*p* < 0.0001), BMI (*p* < 0.0001), waist (*p* < 0.0001), HDL-c (*p* < 0.001; inversely), triglycerides (*p* < 0.01), FPG (*p* < 0.01), HbA_1c_ (*p* < 0.01), UA (*p* < 0.0001), hs-CRP (*p* < 0.05), diabetic medication (*p* < 0.05), and dyslipidemic medication (*p* < 0.01) were significantly associated with VFA areas in females. In multivariate analysis for VFA levels after adjustment for age, sex, and medications for hypertension, diabetes, and dyslipidemia, the significance of weight (*p* < 0.0001), BMI (*p* < 0.0001), and waist (*p* < 0.0001) in both genders were still remained. In order to investigate the impacts of waist and BMI on VFA, we create the hierarchical model stratified two groups of waist and BMI adjustment for age and sex (Figure 3). Compared to the group of smaller waist and normal BMI, VFA was significantly increased (*p* < 0.0001) in the group of larger waist and obese subjects.

**Table 2:** **Univariate analysis for VFA levels as dependent variable**.

	β	SE	*p*-Value
**Males**			
Age	−0.1568	0.4807	0.7451
Weight	2.7065	0.2402	<0.0001
BMI	10.1890	0.7671	<0.0001
Waist	3.5623	0.2567	<0.0001
Systolic BP	0.2806	0.2235	0.2128
Diastolic BP	0.6907	0.4380	0.1186
Total cholesterol	0.1245	0.0906	0.1733
HDL-cholesterol	−0.4610	0.2767	0.0995
LDL-cholesterol	0.1626	0.1159	0.1644
Triglycerides^a^	34.0759	6.7193	<0.0001
FPG	0.3740	0.1409	0.0095
HbA_1c_	0.5024	4.8318	0.0526
Uric acid (UA)	0.2174	3.3067	0.9477
hs-CRP^a^	4.2617	3.1802	0.1839
C-IMT	35.1616	23.8340	0.1439
Hypertensive medication	16.6439	7.6617	0.0326
Diabetic medication	−0.6386	10.9786	0.9538
Dyslipidemic medication	19.9281	9.4195	0.0373
Habitual smoking	−16.2424	9.1387	0.0791
Alcohol intake	20.3688	7.5806	0.0087
**Females**			
Age	0.6573	0.2308	0.0051
Weight	2.1149	0.2131	<0.0001
BMI	6.2002	0.4999	<0.0001
Waist	2.0749	0.1893	<0.0001
Systolic BP	0.2591	0.1233	0.0376
Diastolic BP	−0.1734	0.2701	0.5219
Total cholesterol	−0.0260	0.0730	0.7226
HDL-cholesterol	−0.6418	0.1596	<0.0001
LDL-cholesterol	0.1157	0.0843	0.1722
Triglycerides^a^	17.2461	5.9565	0.0045
FPG	0.7795	0.2358	0.0012
HbA_1c_	13.2390	4.6175	0.0049
UA	10.7302	2.1050	<0.0001
hs-CRP^a^	4.5772	1.8357	0.0139
C-IMT	53.0877	19.5612	0.0076
Hypertensive medication	10.1500	4.9929	0.0441
Diabetic medication	28.8420	10.6327	0.0076
Dyslipidemic medication	19.0611	5.0749	0.0003
Habitual smoking	−17.9344	16.3603	0.2750
Alcohol intake	−7.6024	9.6991	0.4346

*VFA, visceral fat area; BMI, body mass index; BP, blood pressure; HDL, high-density lipoprotein; LDL, low-density lipoprotein; FPG, fasting plasma glucose; hs-CRP, high sensitivity C-reactive protein; C-IMT, carotid intima-media thickness*.

*^a^Log-transformed values were used for the analysis*.

**Table 3:** **Multivariate analysis adjusted for age for VFA levels as dependent variable**.

	β	SE	*p*-Value
**Males**			
Weight	2.8835	0.2405	<0.0001
BMI	10.2088	0.7730	<0.0001
Waist	3.5655	0.2586	<0.0001
Systolic BP	0.3025	0.2279	0.1881
Diastolic BP	0.6924	0.4517	0.1292
Total cholesterol	0.1282	0.0969	0.1896
HDL-cholesterol	−0.1960	0.2824	0.0828
LDL-cholesterol	0.1638	0.1215	0.1813
Triglycerides^a^	34.6199	6.8524	<0.0001
FPG	0.3722	0.1420	0.0105
HbA_1c_	9.4693	4.9282	0.0582
Uric acid (UA)	0.1576	3.3289	0.9623
hs-CRP^a^	4.3902	3.2070	0.1748
C-IMT	38.2550	24.1002	0.1209
Hypertensive medication	21.1059	8.3337	0.0132
Diabetic medication	−0.8550	11.0571	0.9385
Dyslipidemic medication	19.8615	9.4746	0.0391
Habitual smoking	−18.1511	9.4618	0.0585
Alcohol intake	20.3310	7.6233	0.0092
**Females**			
Weight	2.4767	0.1821	<0.0001
BMI	6.2924	0.4589	<0.0001
Waist	2.0154	0.1943	<0.0001
Systolic BP	0.1855	0.1249	0.1399
Diastolic BP	−0.1495	0.2361	0.5709
Total cholesterol	0.0162	0.0727	0.8238
HDL-cholesterol	−0.5591	0.1635	0.0008
LDL-cholesterol	0.1528	0.0825	0.0662
Triglycerides^a^	15.9006	5.8434	0.0074
FPG	0.7485	0.2302	0.0015
HbA_1c_	13.5430	4.4821	0.0030
UA	10.2107	2.0699	<0.0001
hs-CRP^a^	4.2949	1.7938	0.0181
C-IMT	32.7282	23.0059	0.1573
Hypertensive medication	4.8418	5.5088	0.3811
Diabetic medication	26.5890	10.4157	0.0119
Dyslipidemic medication	16.9170	5.0792	0.0011
Habitual smoking	−14.2617	16.0081	0.3747
Alcohol intake	−5.4078	9.4861	0.5696

*VFA, visceral fat area; BMI, body mass index; BP, blood pressure; HDL, high-density lipoprotein; LDL, low-density lipoprotein; FPG, fasting plasma glucose; hs-CRP, high sensitivity C-reactive protein; C-IMT, carotid intima-media thickness*.

*^a^Log-transformed values were used for the analysis*.

Comparison between various parameters stratified by VFA (<100 vs. ≧100 cm^2^) was demonstrated in Table 4. Male gender (*p* < 0.05), weight (*p* < 0.0001), BMI (*p* < 0.0001), waist (*p* < 0.0001), HDL-c (*p* < 0.05; inversely), triglycerides (*p* < 0.0001), FPG (*p* < 0.0001), HbA_1c_ (*p* < 0.01), UA (*p* < 0.01), hs-CRP (*p* < 0.05), and c-IMT (*p* < 0.05) were significantly associated with elevated VFA areas (≧100 cm^2^).

**Table 4:** **Comparison between parameters stratified by VFA**.

	VFA < 100 cm^2^	VFA ≥ 100 cm^2^	*p*-Value
*N*	181	34	
Age (years)	66.9 ± 9.8	66.8 ± 9.1	0.985
Sex (*n*, %male)	68 (37.6)	20 (58.8)	0.017
Weight (kg)	56.1 ± 7.6	73.2 ± 12.4	<0.0001
BMI (kg/m^2^)	23.3 ± 2.5	27.9 ± 3.5	<0.0001
Waist (cm)	83.8 ± 7.8	95.6 ± 9.3	<0.0001
Systolic BP (mmHg)	136.7 ± 18.7	140.1 ± 19.9	0.338
Diastolic BP (mmHg)	85.2 ± 9.0	87.1 ± 9.9	0.264
Total cholesterol (mg/dl)	203.2 ± 36.0	202.2 ± 51.9	0.888
HDL-cholesterol (mg/dl)	62.6 ± 14.9	54.1 ± 16.2	0.003
LDL-cholesterol (mg/dl)	119.1 ± 28.5	120.7 ± 44.9	0.789
Triglycerides (mg/dl)^a^	87.0 ± 44.3	128.1 ± 76.1	<0.0001
FPG (mg/dl)	97.5 ± 17.2	116.9 ± 28.9	<0.0001
HbA_1c_ (NGSP) (%)	5.5 ± 0.6	6.0 ± 0.9	0.001
eGFR (ml/min/1.73 m^2^)	74.2 ± 17.1	73.0 ± 18.5	0.714
Uric acid (mg/dl)	5.1 ± 1.3	5.9 ± 1.2	0.001
hs-CRP (mg/dl)^a^	0.039 ± 0.131	0.069 ± 0.202	0.023
C-IMT (mm)	0.75 ± 0.14	0.81 ± 0.15	0.027

*Data are means ± SDs, geometric mean, range, or percent*.

*VFA, visceral fat area; BMI, body mass index; BP, blood pressure; HDL, high-density lipoprotein; LDL, low-density lipoprotein; FPG, fasting plasma glucose; eGFR, estimated glomerular filtration rate; hs-CRP, high sensitivity C-reactive protein; C-IMT, carotid intima-media thickness*.

*^a^Log-transformed values were used for the analysis*.

To compare the usefulness between VFA and BMI, we performed the multiple stepwise regression analysis. Then, we found that triglycerides (*p* < 0.0001), FPG (*p* = 0.0002), and HDL-c (*p* = 0.0144; inversely) were independently related to VFA (*R*^2^ = 0.2698), whereas; FPG (*p* = 0.0001), HDL-c (*p* < 0.0049; inversely), and age (*p* = 0.0262) were independently related to BMI (*R*^2^ = 0.1375), suggesting that VFA is stronger statistical power to find out not only obese subjects but also subjects with accumulation of visceral fat than BMI.

## Discussion

The adipocytokines secreted by excessive VFA are closely linked with metabolic disorders, such as type 2 diabetes, hypertension, and dyslipidemia, which develop various metabolic and cardiovascular disorders during several decades (25). Therefore, it is important for each individual in general population to recognize VFA for the primary prevention of cerebro-cardiovascular diseases in health checkup examinations, which should be simple and at low cost. In the present study, we have indicated that the VFA measurement by a new BIA method is useful and suitable to general population in the evaluation of adiposity of individuals. Conventional BIA approaches have estimated total fat content but not regional fat distribution (26, 27). Compared to the new method, waist circumference underestimates visceral fat amount because of the accumulation of abdominal subcutaneous fat (28–30). From the results of hierarchical multiple regression, our data may propose that the usefulness of VFA by DUALSCAN shows its superiority over waist and BMI in this study setting. In the multiple stepwise regression analysis, VFA is stronger statistical power to find out not only obese subjects but also subjects with accumulation of visceral fat than BMI. The abdominal dual BIA method of the DUALACAN seemed to measure VFA more precisely with less influence of subcutaneous fat area compared with the conventional whole-body BIA method (31). Because VSA measurement using the DUALSCAN showed a closer correlation with metabolic variables than whole-body BIA method in a health checkup examination, the device was approved as medical device by Health, Labour, and Welfare Ministry since 2011 in Japan (32). Moreover, the evaluation of VFA by the BIA is really a simple and easily accessible device even in a health checkup examination in Japanese general population.

Comparisons between DEXA or abdominal CT and BIA have been already performed in healthy adults (33, 34) and overweight individuals (35). Estimated VFA by BIA was accurate and accessible device is also admitted in obese patients (36, 37). On the contrary, some reports have indicated that the validity, reliability, and accuracy of BIA were not satisfied (38, 39). Although we did not examine the validity and accuracy of BIA, Japanese investigators (40, 41) reported the strong correlations between VFA by abdominal CT and VFA by BIA (*r* = 0.88, *p* < 0.001), between VFA by X-ray CT and VFA by BIA (*r* = 0.89, *p* < 0.001). BIA also has favorable effects on evaluation of skeletal muscle mass (42) and on assessing fat-free mass (43). We consider that VFA measurement by this technique can be enough as a screening to recognize the accumulation of visceral fat, which leads to MetS and increases risks of cerebro-cardiovascular diseases.

VFA levels are significantly associated with metabolic factors such as waist circumference FPG, and triglycerides. These significant associations are remained after adjustment for age and sex. The significance of hs-CRP with VFA deserves consideration. Report from Turkey (44) is consistent with ours. One mechanism for the elevation of hs-CRP in obese individuals might be associated with a high production of cytokines such as IL-6 and TNF-α by the excess adipose tissue, which would induce higher hs-CRP production by the liver (45).

The present study has several limitations. First, this study was cross-sectional with comparatively a small number of subjects. Prospective studies with a large number are needed to investigate the evaluation of role of BIA. Second, our population was relatively healthy, and most of them had BMI within normal limits. Thus, it is necessary to investigate the role of BIA in heterogeneous populations. Third, we do not have the data on the potential bias caused by metabolic factors such as medication and possibility of having edematous diseases. Fourth, although this study was performed in the typical fishing area in Japan, the background of these subjects may be a little different from those of other Japanese people. Fifth, we did not distinguish a clinical importance of VSA from SFA and also did not append the relationship between the SFA and other variables because we did not have data on SFA levels. Finally, we were not able to compare DUALSCAN and CT in the enrolled subjects, because we have no CT scan data.

In conclusion, our results may suggest that a new simple measurement system of visceral fat accumulation by BIA is useful to measure VFA even in a health checkup examination in a general population.

## Author Contributions

ME: conception and design of the study, drafting of the article, and critical revision of the article for important intellectual content. HA: conception and design of the study, drafting of the article. AF: analysis and interpretation of data, EK: analysis and interpretation of data, SN, YN, SK, EN, NM, TT, and AS: collection and assembly of data. YF: final approval of the article.

## Conflict of Interest Statement

The authors declare that the research was conducted in the absence of any commercial or financial relationships that could be construed as a potential conflict of interest.
